# Clinical Characteristics of Intravenous Injection of Monosialotetrahexosyl Ganglioside Sodium-Related Guillain-Barre Syndrome

**DOI:** 10.3389/fneur.2019.00225

**Published:** 2019-03-15

**Authors:** Miao Shi, Jie Zhu, Hui Deng

**Affiliations:** ^1^Department of Neurology, Neuroscience Center, First Hospital of Jilin University, Jilin University, Changchun, China; ^2^Department of Neurobiology, Care Sciences and Society, Karolinska Institute, Stockholm, Sweden

**Keywords:** Guillain-Barre syndrome, monosialotetrahexosyl ganglioside sodium, blood-nerve barrier, GM1-IgG, electrophysiology

## Abstract

**Introduction:** Guillain Barre Syndrome (GBS) is an acute inflammatory immune-mediated multiple nerve root neuropathy. GBS primarily damages the spinal nerve root and peripheral nerves, but can also affect the cranial nerves and cause acute demyelination. This study analyzed the clinical features of intravenous injection of monosialotetrahexosyl ganglioside sodium-related Guillain-Barre syndrome (GRD-GBS).

**Methods:** We retrospectively studied 12 patients who developed GRD-GBS after receiving monosialotetrahexosyl ganglioside sodium treatment in association with recent trauma, surgery, acute cerebrovascular disease, or chronic peripheral neuropathy. Clinical characteristics, electrophysiological examinations, serum-specific antibodies, and prognosis were assessed. As controls, we selected 12 patients hospitalized with non-ganglioside-related (NGRD)-GBS.

**Results:** The positive rate of the ganglioside antibody test was significantly higher in the GRD-GBS group (66.67%) than in the NGRD-GBS group (8.33%). CSF protein levels were similar between the groups, but the incidence of blood-nerve-barrier (BNB) disruption was higher in the GRD-GBS group. Patient scores for the Hughes Functional Grading Scale (HFGS), a disability scale, were higher (more severe disability) in the GRD-GBS group than in the NGRD-GBS group. The HFGS scores of the GRD-GBS group did not change between peak onset and 30 days after discharge, but did change significantly by 90 days after discharge, while scores were significantly lower at both 30 and 90 days after discharge in the NGRD-GBS group.

**Conclusions:** GRD-GBS patients showed more severe clinical manifestations, poorer prognosis, and slower recovery than patients with NGRD-GBS. Ganglioside treatment should be used with extreme caution in patients with trauma that damages the BNB.

## Introduction

Guillain Barre Syndrome (GBS) is an acute inflammatory immune-mediated multiple nerve root neuropathy (1). GBS primarily damages the spinal nerve root and peripheral nerves but can also affect the cranial nerves and cause acute demyelination (2). Most clinical symptoms of GBS are acute onset, the disease progresses gradually, and the disease peaks at about 2 weeks after onset. The disease manifests as multiple nerve root and peripheral nerve lesions, and cerebrospinal fluid (CSF) examination shows a phenomenon of protein-cell separation. GBS is primarily a single-phase disease, and intravenous immunoglobulin (IVIG) treatment is effective.

At present, the etiology of GBS is unclear. However, studies have reported that the incidence may be related to *Campylobacter jejuni* (CJ) infection (3). Further, several studies have reported that the risk of GBS increases after surgery (4–6) and some have shown that the incidence of postoperative GBS was significantly higher than the incidence associated with the flu vaccine and other infectious factors that had been previously linked to GBS (4). Recently, our hospital has admitted patients who developed GBS after application of monosialotetrahexosyl ganglioside sodium as part of a treatment associated with recent trauma, surgery, acute cerebrovascular disease, or chronic peripheral nerve disease. The component of monosialotetraose ganglioside drug, which is extracted from pig brain and has a function on nerve cell function damage, is still used in many hospitals in China. This study analyzed the clinical features of intravenous injection of monosialotetrahexosyl ganglioside sodium-related Guillain-Barre syndrome (GRD-GBS) and reports possible mechanisms of this disease based on our findings.

## Materials and Methods

### Subjects

This was a retrospective study. This study has been approved by the ethics committee of the First Hospital of Jilin University, Changchun, China, and was performed in accordance with the ethical standards laid down in the 1964 Declaration of Helsinki and its later amendments. Written informed consent was obtained from all patients. Participants included 12 patients with GRD-GBS who had received ganglioside (single sialic acid ganglioside, cerebroside peptide) treatment in association with recent trauma, surgery, acute cerebrovascular disease, or chronic peripheral neuropathy between January and November 2017 at the department of Neurology of the First Hospital of Jilin University, Changchun, China. The control group consisted of 12 patients with non-ganglioside-related (NGRD)-GBS hospitalized during the same period. All 24 patients met the diagnostic criteria for GBS (7–10).

The inclusion criteria for the NGRD-GBS group were (1) first occurrence of GBS for a given patient; (2) received a ganglioside antibody test; and (3) received follow-up. Exclusion criteria included specific types of GBS, such as Miller-Fisher Syndrome, that could affect the comparison between the two groups of patients due to different clinical manifestations and laboratory test results typical in these sub-types of GBS.

Clinical data for each patient were retrospectively analyzed, including basic information, disease course, form of onset, clinical manifestations, electrophysiology, CSF analysis, serum GM1-IgG, treatment, and prognosis.

### Evaluation of Clinical Severity

The Hughes Functional Grading Scale (HFGS), which provides a measure of disability (11, 12), was used to rate clinical performance. Motor function deficits were scored on the HFGS scale, ranging from 0 to 6 (Table 1), with higher numbers indicating more severe disability.

**Table 1:** Hughes functional grading scale.

**Hughes score**	**Clinical performance**
0	Normal
1	Slight clinical symptoms and signs
2	Able to walk 5 m or more without assistance but unable to run
3	Able to walk 5 m with help
4	Bedridden or chair-bound
5	Ventilator-assisted breathing
6	Death

### Laboratory Examinations

All patients in both groups received a neuroelectrophysiological examination, including electrophysiology and electroneurography, 14 days after onset of clinical symptoms. The sensory fibers (ruler, median, superficial, and sural nerves) and motor fibers (ruler, median, hernia, and peroneal nerves) from 4 nerves of the upper and lower extremities were examined in each patient; a total of 96 nerves were sampled for each group. The latency, amplitude, and conduction velocity of the sensory and motor fibers were measured and evaluated for normalcy according to electrophysiological diagnostic criteria proposed by Rajabally (13) to indicate myelin or axonal injury. Lumbar puncture was performed to detect CSF protein levels. Serum anti-ganglioside antibodies GM1-IgG were detected using ELISA (Euromont Kit) (14).

### Statistical Analysis

Statistical analysis was performed using SPSS version 22.0 software. The normal distribution measurement data are represented as mean ± standard deviation; student *t*-test was used for comparison of these data between the groups. Median (P25, P75) was used for non-normal distribution measurement data, and rank sum test was used for comparison between groups. The count data are expressed as percentage; Chi-square or Fisher exact tests were used for comparison of these data between groups. For all statistical tests, *P* < 0.05 indicated statistical significance.

## Results

### Clinical Data

There was no difference in age or hospital stay duration between the two groups [GRD-GBS group: age, 59.58 ± 5.50 years; hospital stay duration, 18.00 (10.75, 37.50) days; NGRD-GBS: age, 54.83 ± 12.87 years; hospital stay duration, 15.50 (13.25, 20.25) days], however the GRD-GBS group had a 1:1 male to female ratio, while this ratio was 2:1 for the NGRD-GBS group. For the GRD-GBS group, limb weakness was the first symptom in 100.00% of the cases, accompanied by numbness in 4 cases (33.33%), respiratory muscle weakness in 3 cases (25.00%), symptoms of cranial nerve damage in 1 case (8.33%), and nerve trunk pain in extremities in 3 cases (25.00%). In the NGRD-GBS group, limb weakness was the first symptom in 11 cases (91.67%), accompanied by limb numbness in 8 cases (66.67%), respiratory muscle weakness in 2 cases (16.67%), symptoms of cranial nerve damage in 2 cases (16.67%), and nerve trunk pain of sleeves in 5 cases (41.67%). In one case, limb numbness was the first symptom (8.33%).

All patients in the GRD-GBS group were administered venous monosialotetrahexosyl ganglioside sodium as part of treatment for a premorbid event, and the latency from monosialotetrahexosyl ganglioside sodium-treatment to symptom onset was 14.64 ± 7.87 days; premorbid events included lumbar vertebrae surgery in 3 cases (all 3 cases involved lumbar posterior discectomy, spinal decompression, bone graft fusion, and internal fixation), cervical vertebral surgery in 1 case (cervical spinal stenosis by spinal decompression), tenosynovitis surgery in 1 case, cerebrovascular disease in 4 cases (3 cases of cerebral infarction and 1 case of cerebral hemorrhage), trauma in 2 cases (upper extremity fracture in 1 case and multiple thoracolumbar fracture in 1 case), and chronic peripheral neuropathy in 1 case. Prodromic infections of the NGRD-GBS group included respiratory tract infection in 6 patients (50%) and gastrointestinal infection in 1 patient (8.33%). Of the remaining patients in the NGRD-GBS group, 1 patient had surgical treatment of parotid cysts and cataracts (8.33%) and 4 patients had no related pre-illness conditions (33.33%). Therefore, there were 8 cases (66.67%) in the blood-nerve barrier disruption of GRD-GBS and 1 case (8.33%) in the NGRD-GBS group, blood-nerve barrier destruction rate were higher in the GRD-GBS group (66.67%) than in the NGRD-GBS group (8.33%) (*P* < 0.05).

Approximately 2 weeks after GBS onset, a total of 10 patients in the GRD-GBS group received lumbar puncture (2 patients were not suitable for lumbar puncture); 9 patients had CSF protein-cell separation (91.67%) and the mean CSF protein content for the patients who received lumbar puncture was 0.935 (0.580, 1.548) g/L. All 12 patients in the NGRD-GBS group also received lumbar puncture approximately 2 weeks after GBS onset; 9 patients had CSF protein-cell separation (75%) and the mean CSF protein content was 0.640 (0.430, 1.727) g/L. CSF protein levels showed no difference between the two groups (*P* > 0.05). The ganglioside antibody test showed that the positive rate in the GRD-GBS group was 66.67%, and the positive rate in the NGRD-GBS group was 8.33% (*P* < 0.05).

EMG was performed 2 wk following onset of GBS. According to Rajabally's criteria, 11 patients belong to the AMAN type (91.67%), and the remaining 1 patient is the AIDP type (9.09%) in the GRD-GBS group; in the NGRD-GBS group, 5 patients are the AMAN type (41.67%), 4 patients show the AIDP type (33.33%), and 3 patients show normal myelination (25%). Electrophysiology examination showed significantly difference about AMAN type between the GRD-GBS group and NGRD-GBS group (*P* < 0.05).

### Treatment and Prognosis

Both groups of patients were treated with gamma globulin (0.4 g/kg × 5 d). In the GRD-GBS group, 3 patients were treated with glucocorticoid therapy (methylprednisolone 80 mg/d, with gradually reduced dosages and discontinued use after about 1 month) and 3 patients received ventilator assistance due to respiratory muscle involvement. In the NGRD-GBS group, 2 patients received glucocorticoid therapy (same treatment plan as the GRD-GBS group) and 2 patients received ventilator assistance due to respiratory muscle involvement. There was no significant difference in respiratory muscle paralysis and number of hospitalization days between the two groups (*P* > 0.05). Three patients in the GRD-GBS group were hospitalized for dyspnea 2 times and 2 patients died after discharge, who died of respiratory failure.

HFGS scores at the time of peak disease, 30 days, and 90 days after discharge in the GRD-GBS group were 4.00 (4.00, 4.75), 4.00 (4.00, 4.00), and 3.00 (2.00, 4.00), respectively. These scores in the NGRD-GBS group were 4.00 (3.00, 4.00), 1.50 (1.00, 3.00), and 0.50 (0.00, 1.00), respectively. HFGS scores were higher in the GRD-GBS group than in the NGRD-GBS group (*P* < 0.05). In the GRD-GBS group, there was no significant change in the HFGS scores at 30 days after discharge compared with those at peak onset (*P* > 0.05), however significant improvement was observed at 90 days after discharge (*P* < 0.05). In contrast, the HFGS scores of peak onset in NGRD-GBS group were significantly higher than those at 30 and 90 days after discharge (*P* < 0.01).

## Discussion

GBS includes acute inflammatory demyelinating polyradiculoneuropathy (AIDP), acute motor axonal neuropathy (AMAN), acute motor-sensory axonal neuropathy (AMSAN), Miller-Fisher syndrome (MFS), and certain other rare subtypes (15). AMAN and AMSAN are generally believed to be caused by ganglioside antibodies infiltrating nodes of Ranvier (16). The predominant clinical features of AIDP (17) are symplectic soft paralysis of extremities, which can be associated with diminished pain in the distal extremities; a lack of specific antigen-antibody markers; an electromyogram primarily characterized by decreased conduction velocity (10); and pathology showing peripheral nerve segmental demyelination. AMAN is a severe condition with frequent respiratory system involvement and ventilator dependence (17). Even with only mild cranial and autonomic nervous system involvement, AMAN is almost purely a motor syndrome, with rapid progression of limb weakness, and sensory nerves are rarely or only slightly involved. GM1-IgG is a characteristic biomarker of AMAN; the predominant electromyogram feature is amplitude reduction, with or without conduction block (18); and motor axonal damage is evident upon pathological assessment.

In the GRD-GBS group, 7 of 12 patients had trauma or surgery prior to onset. The relationship between surgery and GBS is unclear. Gensicke et al. (4) reported that 6 patients (9.5%) with GBS had undergone surgery in a 6-week period before onset of GBS symptoms and suggested that the relative risk of developing GBS within the 6 weeks after surgery is 13.1 times higher than that of the general population. However, another report has suggested that this risk is not increased by the surgery itself but rather by a transient immunosuppression after the operation, which increases the risk of infection, both clinical and subclinical, and may increase the risk of postoperative GBS (6). Brown and Snow (19) reported that GBS is more likely to occur in patients with blood-nerve-barrier impairment. All the seven surgery patients in the study presented here had varying degrees of damage to the blood-nerve barrier. Damage to this innate protective barrier enables the entry of many potential antigen components from the blood into the nervous system, where they can induce cross-reactive immune responses. Of note, four of the patients in the GRD-GBS group experienced strokes as their premorbid event. Candelise and Ciccone (20) reported that in 2,265 patients of cerebral vascular diseases involving treatment with gangliosides from 2000 to 2002, only eight patients had adverse reactions, one of whom developed GBS. Therefore, further research is required to explore the relationship between GBS and cerebrovascular disease.

In the study presented here, all 12 patients with GRD-GBS had received monosialotetraose ganglioside intravenously, around 2 weeks before the onset of GBS, in association with recent surgery, trauma, acute stroke, or chronic peripheral neuropathy. The relationship between the onset of GBS and the use of monosialotetraose ganglioside treatment has been controversial. Three surveys in Italy's Ferrara region (21) showed that ganglioside therapy did not increase the risk of GBS and that the incidence of GBS did not change after ganglioside withdrawal. Therefore, Govoni et al. (21) suggested that the occurrence of GBS after application of monosialotetraose ganglioside may be related to the susceptibility of the individuals receiving the ganglioside treatment. However, the study by Latov et al. (22) was the first report of ganglioside-induced GBS and, since then, cases of GBS have been reported with injection of gangliosides for the treatment of sciatica, stroke, and others (23). Laboratory studies have also shown that the use of gangliosides induces an animal model of GBS (14, 24). Additionally, Yuki et al. (25) successfully established an animal of AMAN in Japanese white rabbits using bovine brain ganglioside. The serum from this AMAN model showed high anti-GM1-IgG antibody titers, and the disease manifested as sudden limb weakness and was a monophasic disease.

Pathogenesis of GBS may occur via macrophages in the peripheral blood, which recognize and bind to bacterial protein antigens and then activate T cells (16). Certain activated T cells can cross the damaged BNB and release cytokines, such as TNF-α and IFN-γ, which activate macrophages in the endoneurium and interact with toxic nitric oxide (NO) radicals, both of which then invade and destroy dense myelin (16). Another fraction of activated T cells releases IL-4, IL-6, and other cytokines, thereby stimulating B cells to produce antibodies that can cross the damaged BNB where they cooperate with complement C5-9 to destroy Schwann cells and thus produce vesicular dissolution of myelin, causing AIDP (16). Further, when peripheral blood macrophages recognize and bind to ganglioside-like bacterial antigens, they directly stimulate B cells to produce antibodies that can cross a damaged BNB and cooperate with complement C5-9 to block conduction or cause axonal degeneration, causing AMAN (16). Further, this previous study showed that in AMAN, a ganglioside antibody–associated GBS, axonal damage was more serious than in AIDP and that exogenous gangliosides were immunogenic and triggered production of anti-ganglioside antibodies in nerve endings, especially IgG antibodies, leading to self-immune demyelination or axonal degeneration (23). These findings are similar to those of previous studies.

In the study presented here, the 12 patients with GRD-GBS had recently been treated with monosialotetraose ganglioside in association with trauma, surgery, acute cerebrovascular disease, or chronic peripheral nerve disease, resulting in GBS. These patients had stronger peak symptoms than did patients in the NGRD-GBS group. Further, the motor nerve injury was more serious in the GRD-GBS group than in the NGRD-GBS group, especially injury of the motor nerve axons. This discrepancy could be due to the presence of blood-nerve barrier disruption in the GRD-GBS group. Specifically, monosialotetraose ganglioside in the serum may have entered the nervous system through the disrupted BNB and then stimulated B cell production of ganglioside antibodies, which ultimately blocked conduction or induced axonal damage. These events could have caused destruction of myelin sheaths and axons and the eventual occurrence of GBS, which was dominated by the presentation of extensive motor axonal lesions.

GRD-GBS is a rapidly progressive and severe disease. Of the patients in our GRD-GBS study group, 3 were hospitalized twice for dyspnea and 2 died after discharge. The HFGS scores of the GRD-GBS group did not change between peak onset and 30 days after discharge, yet did change significantly by 90 days after discharge. Thus, these patients had poor prognosis. Contrarily, the HFGS scores in the NGRD-GBS group were significantly reduced at 30 and 90 days after discharge. Specifically, these patients showed obvious recovery regarding limb paralysis when compared with their onset of disease and had good prognosis. Thus, compared with the NGRD-GBS group, the GRD-GBS group had more severe clinical manifestations, slower recovery, and worse prognosis. Therefore, when unexplained progressive weakness occurs after use of monosialotetraose ganglioside therapy, the possibility of GBS must be considered and appropriate measures taken to relieve or delay onset of GBS symptoms.

## Conclusion

When a patient has damage to the barrier between the circulatory system and the nervous system, due to trauma, surgery (especially cervical, lumbar, and limbs surgeries), and cerebrovascular diseases, ganglioside drugs should be used with caution, as GBS may be easily induced and, in such GBS cases, the patient's clinical manifestations will likely be severe, namely, the occurrence of AMAN type GBS. Electromyograms reveal that damage occurs predominantly in motor axons, and patients have poor prognosis and slow recovery.

The limitation of this study is that all patients in the GRD-GBS group developed GBS after treatment for surgery, trauma, cerebrovascular disease, and peripheral neuropathy. Patients who had also experienced these associated conditions/procedures and did not develop GBS were not included or used for comparisons in our study.

## Data Availability

Datasets are available per request to the corresponding author.

## Author Contributions

All authors listed have made a substantial, direct and intellectual contribution to the work, and approved it for publication.

### Conflict of Interest Statement

The authors declare that the research was conducted in the absence of any commercial or financial relationships that could be construed as a potential conflict of interest.
